# Porosity Engineering towards Nitrogen-Rich Carbon Host Enables Ultrahigh Capacity Sulfur Cathode for Room Temperature Potassium–Sulfur Batteries

**DOI:** 10.3390/nano12223968

**Published:** 2022-11-10

**Authors:** Jingzhe Liang, Wanqing Song, Haozhi Wang, Jia Ding, Wenbin Hu

**Affiliations:** 1 School of Materials Science and Engineering, Tianjin Key Laboratory of Composite and Functional Materials, Key Laboratory of Advanced Ceramics and Machining Technology (Ministry of Education), Tianjin University, Tianjin 300072, China; 2 Joint School of National University of Singapore and Tianjin University, International Campus of Tianjin University, Binhai New City, Fuzhou 350207, China

**Keywords:** potassium–sulfur battery, porosity engineering, nitrogen-rich carbon hosts, potassiation kinetics, density functional theory

## Abstract

Potassium–sulfur batteries (KSBs) are regarded as a promising large-scale energy storage technology, owing to the high theoretical specific capacity and intrinsically low cost. However, the commercialization of KSBs is hampered by the low sulfur utilization and notorious shuttle effect. Herein, we employ a porosity engineering strategy to design nitrogen-rich carbon foam as an efficient sulfur host. The tremendous micropores magnify the chemical interaction between sulfur species and the polar nitrogen functionalities decorated carbon surface, which significantly improve the sulfur utilization and conversion. Meanwhile, the abundant mesopores provide ample spaces, accommodating the large volume changes of sulfur upon reversible potassation. Resultantly, the constructed sulfur cathode delivers an ultrahigh initial reversible capacity of 1470 mAh g^−1^ (87.76% of theoretical capacity) and a superior rate capacity of 560 mAh g^−1^ at 2 C. Reaching the K_2_S phase in potassiation is the essential reason for obtaining the ultrahigh capacity. Nonetheless, systematic kinetics analyses demonstrate that the K_2_S involved depotassiation deteriorates the charge kinetics. The density functional theory (DFT) calculation revealed that the nitrogen-rich micropore surface facilitated the sulfur reduction for K_2_S but created a higher energy barrier for the K_2_S decomposition, which explained the discrepancy in kinetics modification effect produced by the porosity engineering.

## 1. Introduction

Pursuit for high energy density and low-cost electrode materials is of great significance in developing efficient electrochemical energy conversion devices [1,2,3,4]. Alkali-metal–sulfur batteries (LSBs, NSBs, KSBs) have attracted tremendous attention because of the high theoretical specific capacity (1675 mAh g^−1^), low cost, and environmental friendliness of the sulfur cathode [5,6,7,8,9,10]. Nevertheless, due to the scarceness and exorbitant price of Li metal, NSBs and KSBs are more suitable for the large-scale energy storage applications [11,12,13,14,15,16]. High-temperature (HT) NSBs have been demonstrated since the 1960s and have been successfully commercialized over the decades. However, the safety problems caused by the high temperature (300–350 °C) and the limited theoretical capacity (557 mAh g^−1^) limit its further development [2,17]. Consequently, it is urgent to develop room- temperature (RT) NSBs and KSBs. For the more negative redox potential of metal K (K+/K = −2.93 V vs. SHE) than that of metal Na (Na+/Na = −2.71 V vs. SHE), a potassium-based system is expected to deliver higher operating voltage, which is beneficial for the high energy density of practical devices [18,19,20,21]. Recently, researchers have been increasingly pursuing batteries with high active material loading and high energy density to meet the requirements in practical devices. Designing freestanding cathodes to reduce inactive binders or collectors to increase weight capacity and energy density is an effective method [22]. For instance, a freestanding sulfur cathode designed by Lee et al. can achieve ultrahigh sulfur areal loading (7 mg cm^−2^) by optimizing the precursor composition, which facilitates the applicability in large-scale fabrication [23].

Like the LSBs and NSBs, KSBs also face several critical problems [24,25,26,27]. First, the KSBs share the same issues of low electrical conductivity of sulfur (5 × 10^−30^ S cm^−1^) and the severe volume expansion (up to 300%) of LSBs and NSBs. Second, analogous to LSBs and NSBs, long-chain potassium polysulfides (KPSs) can be dissolved in electrolytes, especially ether-based electrolytes. Soluble KPSs will shuttle to the anode side, leading to the loss of active material, rapid capacity decay, and poor cycle stability, which is the notorious shuttle effect. Moreover, the K_2_S_3_ phase with low solubility in ether-based electrolytes is easily deposited and the further potassiation of K_2_S_3_ is a solid-phase reaction [28]. This makes it easier for the fast accumulation of insoluble K_2_S_2_ and K_2_S on the cathode, causing sluggish kinetics and terminating the discharge process with high overpotential [28]. All of these issues clearly indicate that KSBs still face enormous challenges.

To address the aforementioned issues, the design of cathode materials for KSBs is critical. Similar to LSBs and NSBs, various carbonaceous materials with excellent electrical conductivity, precisely controlled porosity, low cost, and simple preparation have been used to host sulfur for KSBs [29,30]. The design strategies for carbon matrix mainly focus on chemical or physical constraints on sulfur species [24,31]. Chemical constraints of sulfur species mainly focus on doping of heteroatoms (N [32], O [33], P [34], B [35], etc.) in carbon matrix and the formation of covalent bonds between sulfur and carbon matrix, as in the case of the pyrolyzed polyacrylonitrile/sulfur nanocomposite (SPAN) [36,37]. Sulfur species are anchored to the carbon matrix by strong chemical adsorption, which can effectively mitigate shuttle effect. As for the physical constraints of sulfur species, the main focus is on designing diverse nanostructures of the carbon matrix, such as delicately designed carbon nanofibers [38] (1D), carbon nanosheets [39] (2D), carbon nanospheres [40] (3D), and regulating the porosity of carbon matrix, for example, microporous carbon and hierarchical porous carbon [41,42]. The physical adsorption between carbon matrix and sulfur species can be improved by nanostructure design, so that sulfur species are somewhat confined to the cathode side. Research on KSBs cathode materials is still in its infancy stage. In particular, the effects of porosity structure of the carbon host on the K-S redox mechanisms and kinetics have rarely been explored in previous research.

Herein, we employ a porosity engineering strategy towards nitrogen-rich carbon foam as a distinguished sulfur host (named P-NCF). The P-NCF with abundant porosity was created by pre-pyrolysis lyophilization and post-pyrolysis CO_2_ activation. The enormous micropores enhance the chemical interaction between sulfur species and carbon host decorated with polar nitrogen functionalities, which greatly facilitate the utilization and conversion of sulfur. Furthermore, the rich mesopores provide sufficient spaces to accommodate the huge volume expansion of sulfur during reversible potassation. The S@P-NCF cathode delivers excellent electrochemical performances with an ultrahigh reversible specific capacity of 1470 mAh g^−1^ (87.76% of theoretical capacity) and a superior rate capacity of 560 mAh g^−1^ at 2 C. More K_2_S phase in the discharge product is the reason for the ultrahigh capacity achieved by S@P-NCF. The kinetics analyses show that S@P-NCF exhibits superior discharge kinetics but inferior charge kinetics. DFT calculation suggested that the nitrogen-rich micropore surface boosted the formation of K_2_S but simultaneously produced a higher energy barrier for the decomposition of K_2_S, which explains the different modification effect towards K-S redox kinetics by porosity engineering.

## 2. Result and Discussion

**Figure 1:**
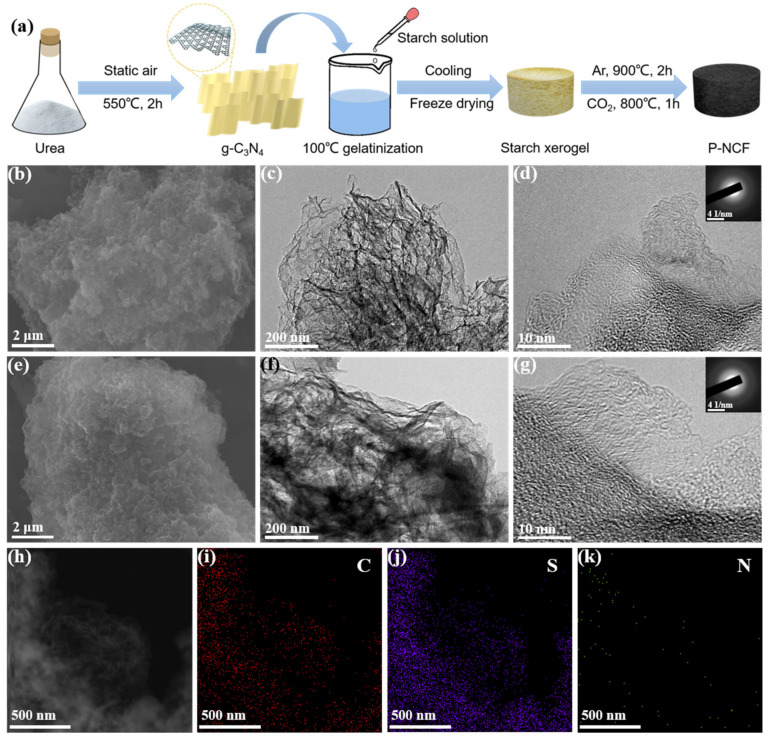
(**a**) Schematic illustration of the synthesis process of P-NCF. (**b**,**e**) SEM and (**c**,**d**,**f**,**g**) TEM images of (**b**–**d**) P-NCF and (**e**–**g**) S@P-NCF. The insets in (**d**,**g**) are the corresponding SAED images. (**h**) Annular dark-field STEM image and (**i**–**k**) corresponding elemental mappings of S@P-NCF.

The introduction of g-C_3_N_4_ as both a nitrogen source and sacrificial template in the starch gelatinization enables the preparation of nitrogen-doped carbon materials with abundant pores. Following this methodology, nitrogen-rich carbon foam (NCF) based on porosity engineering strategy (P-NCF) was synthesized and the schematic process is summarized in Figure 1a. First, the g-C_3_N_4_ was obtained via pyrolysis of urea in static air [43]. During the subsequent high-temperature carbonization, g-C_3_N_4_ degrades automatically and creates porous structures and nitrogen heteroatoms in the carbon matrix [44]. The g-C_3_N_4_ was dispersed in deionized water and heated to 100 °C under vigorous stirring. Subsequently, the starch solution was added into the aforementioned mixture under continuous stirring, and the mixture gradually thickened. After the starch gelatinization process, the viscous mixture is cooled to room temperature and freeze dried to obtain starch xerogel. Subsequently, the starch xerogel was carbonized at 900 °C under Ar atmosphere, and then activated at 800 °C under CO_2_ atmosphere to form the final product P-NCF. In contrast, the sample without CO_2_ activation is named as NCF. The final cathode materials S@P-NCF and S@NCF were obtained by an initial sulfur vapor-infiltrated method at 500 °C and a following heat treatment at 200 °C to remove the surficial sulfur. The morphologies of the carbon hosts and sulfur-impregnated composites were characterized by scanning electron microscopy (SEM) and transmission electron microscopy (TEM). As shown in Figure 1b–g, Figures S1 and S2, all samples exhibit a macro-morphology, which consists of graphene-like flexible ultrathin nanosheets. The structure stability of P-NCF/NCF as a sulfur host is verified by the unchanged macro-morphology after sulfur loading. There are no agglomerated sulfur particles in Figure 1e–g and Figure S2, indicating the complete penetration of sulfur into the porosity of carbon hosts. As further evidence, the energy-dispersive X-ray spectroscopy (EDS) mappings show a uniform distribution of sulfur signal in P-NCF (Figure 1h–j). Notably, in the selected area electron diffraction (SAED) patterns (Figure 1g inset and Figure S2c inset), only diffuse diffraction rings are observed, suggesting the amorphous texture of sulfur in the composites. The nitrogen signal overlaps well with the carbon matrix, indicating a nitrogen-rich surface chemistry of the sulfur host. The nitrogen groups enhance the polarity of the carbon surface, which increase the affinity between the sulfur species and carbon host [45].

**Figure 2:**
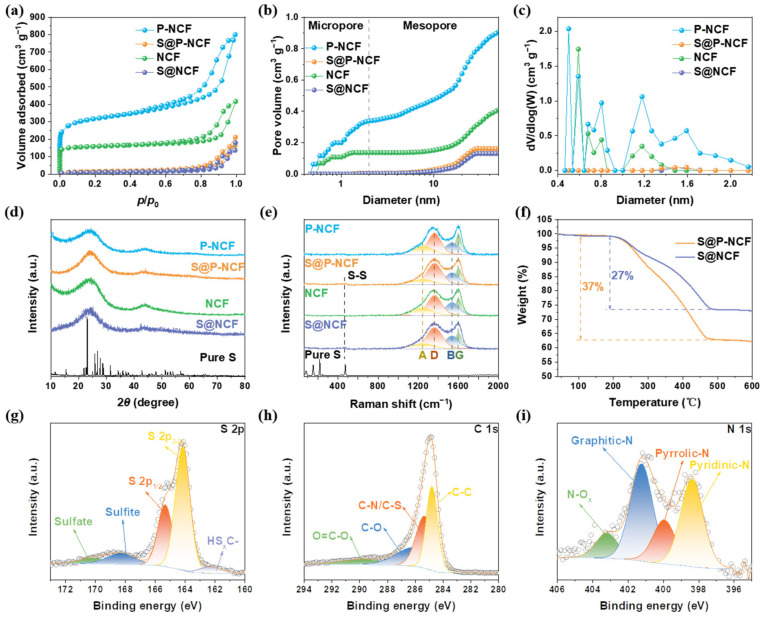
(**a**) Nitrogen adsorption–desorption isotherms, (**b**) cumulative pore volumes, and (**c**) pore size distributions of P-NCF, S@P-NCF, NCF, and S@NCF. (**d**) XRD patterns and (**e**) Raman spectra of P-NCF, S@P-NCF, NCF, S@NCF, and pure S. (**f**) TGA curves of S@P-NCF and S@NCF. High-resolution XPS spectra of (**g**) S 2p, (**h**) C 1s for S@P-NCF, and (**i**) N 1s for P-NCF.

The electrochemical activity of sulfur is significantly affected by the molecular structure, which is closely related to the pore structure of the carbon host [46]. Micropores are promising reservoirs to accommodate small-molecule sulfur and avoid the formation of long-chain KPSs [47]. The pore structures of P-NCF, S@P-NCF, NCF, and S@NCF were analyzed by nitrogen adsorption–desorption isotherms (Figure 2a). A large quantity of adsorption at low *P*/*P*_0_ and an obvious hysteresis loop at medium *P*/*P*_0_ indicate the existence of micropores and mesopores, respectively. The Brunauer−Emmett−Teller (BET) surface areas of P-NCF and NCF are 1167.41 and 619.95 m^2^ g^−1^, respectively (Table S1). According to the cumulative pore volumes in Figure 2b, P-NCF has much higher micropore volume (0.34 cm^3^ g^−1^) than NCF (0.14 cm^3^ g^−1^). Figure 2c show the pore size distributions of P-NCF, S@P-NCF, NCF, and S@NCF. More importantly, in addition to the microporosity at 0.6 nm, 0.8 nm, and 1.2 nm, there is extra microporosity with a size of 0.5 nm for P-NCF, which is absent for NCF (Figure 2c). The mesopore volume of P-NCF is also higher than that of NCF. The larger pore volume of P-NCF and the newly created micropores with a diameter of 0.5 nm indicate the intensive porosity generation effect of CO_2_ activation process. Evidently, after sulfur loading, the BET surface area and pore volume of both samples sharply reduced (Table S1). The dramatic decrease in adsorption at low *P*/*P*_0_ indicates that sulfur occupies the micropores of the carbon matrices. The apparent hysteresis loops at medium *P*/*P*_0_ indicate that some mesopores still exist in S@P-NCF and S@NCF. The above-mentioned results show that sulfur occupied almost all micropores in the carbon matrix. Due to the space confinement of the micropores, the sulfur encapsulated in the micropores can only exist as small molecules of sulfur, which is expected to reduce the production of long-chain KPSs and inhibit the shuttle effect [48,49]. Furthermore, the abundant mesopores could accommodate huge volume expansion of sulfur during the reversible potassiation [50]. These advantages are all attributed to the pore engineering of the carbon hosts.

The X-ray powder diffraction (XRD) patterns of carbon hosts and composites are presented in Figure 2d. All samples display two broadened shoulder peaks at 2θ ≈ 24° and 44°, corresponding to the carbon phase. Notably, there are no diffraction peaks of sulfur in S@P-NCF and S@NCF patterns, indicating the amorphous form of sulfur, which is in line with the SAED. As shown in Figure 2e, the Raman spectra were fitted by four Lorentzian peaks: disorder sp^3^ (A), disorder (D), amorphous (B), and graphitic (G) [51]. The area ratio of peak D to peak G (*I*_D_/*I*_G_) can reflect the graphitization degree of carbon [52]. The *I*_D_/*I*_G_ values of P-NCF, S@P-NCF, NCF, and S@NCF are 2.26, 2.89, 2.57, and 3.08, respectively (Figure S3). The lower *I*_D_/*I*_G_ value of P-NCF indicates that CO_2_ is more likely to react with the higher energy disordered carbon during the activation process, thereby increasing the graphitization degree [53]. Increased graphitization induces higher electronic conductivity, which is favorable for the charge transfer in sulfur redox [54]. In addition, there is a weak peak located at ~460 cm^−1^, which is attributed to the S-S stretching [55]. As shown in the TGA data in Figure 2f, the sulfur contents in S@P-NCF and S@NCF are calculated to be 37% and 27%, respectively. The higher sulfur content of S@P-NCF is strong evidence for the improved affinity between sulfur species and P-NCF host via porosity engineering, which restrains the sulfur loss during high temperature sulfur peregrination procedure (500 °C, much higher than routine 155 °C for sulfur loading). The much higher surface area provided by the largely increased porosity can significantly amplify the chemical interaction between the sulfur and polar nitrogen functionalities’ decorated carbon host. Apart from inhibiting sulfur loss during high temperature, the intensified sulfur host affinity is also favorable for the alleviation of electrochemical shuttle effect in the KSBs.

The composition and surface chemical states were further investigated by X-ray photoelectron spectroscopy (XPS). The peaks of O, N, C, and S elements are observed in the survey XPS spectra of S@P-NCF and S@NCF (Figure S4a). High-resolution XPS spectra of S and C elements for S@P-NCF are illustrated in Figure 2g and h, respectively. As shown in Figure 2g, two strong peaks located at 165.3 and 164.1 eV correspond to the S 2p_1/2_ and S 2p_3/2_. The small peak at 162.4 eV indicates a partial charge transfer between the carbon matrix and sulfur [49], which contributes to the sulfur anchoring. In addition, two peaks at the high binding energies of 170.2 and 168.3 eV are ascribed to the presence of sulfate and sulfite [56]. The peak located at 286.1 eV in the C 1s spectrum (Figure 2h) is attributed to the C-N/C-S bond [57], demonstrating the chemical bonding-induced strong affinity between the carbon surface and sulfur. High-resolution XPS spectra of S and C elements for S@NCF (Figure S4b,c) are similar to those for S@P-NCF, which proves that the analogous surface chemistry of S@NCF to S@P-NCF. In addition, high-resolution XPS spectra of N 1s for P-NCF and NCF are shown in Figure 2i and Figure S4d, respectively. The atom percent of nitrogen are 3.5 at% for P-NCF and 3.06 at% for NCF. In particular, in the N 1s XPS spectrum of P-NCF, peaks located at 401.2, 400.0, and 398.4 eV can be assigned to graphitic-N, pyrrolic-N, and pyridinic-N, respectively [58]. The contents of the three types of nitrogen heteroatoms in P-NCF and NCF are shown in Figure S5. Compared to NCF, the proportion of graphitic-N is lower and the proportion of pyrrolic-N and pyridinic-N are higher in P-NCF. It is known that pyridinic-N and pyrrolic-N exhibit relatively stronger interactions with sulfur species [59]. Therefore, the higher proportion of pyridinic-N and pyrrolic-N in P-NCF is one of the underlying reasons for the stronger affinity toward sulfur.

**Figure 3:**
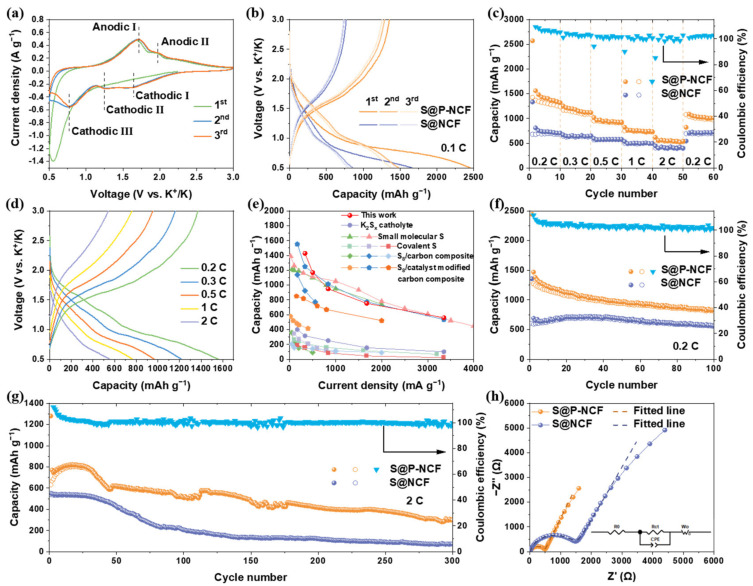
(**a**) CV curves of S@P-NCF cathode at a scan rate of 0.1 mV s^−1^. (**b**) Galvanostatic charge/discharge profiles of S@P-NCF and S@NCF in the first three cycles at 0.1 C. (**c**) Rate performance of S@P-NCF and S@NCF at various current densities. (**d**) Galvanostatic discharge/charge profiles of S@P-NCF at various current densities. (**e**) A comparison of the rate performance of S@P-NCF with recently reported cathodes for KSBs. (**f**) Cycling performance of S@P-NCF and S@NCF at 0.2 C. (**g**) Long-term cycle stability of S@P-NCF and S@NCF at 2 C over 300 cycles. (**h**) Nyquist plots and corresponding fitted lines of S@P-NCF and S@NCF after the initial cycle; the inset is the equivalent circuit model.

In order to examine the electrochemical properties of the S@P-NCF cathode, K-S coin cells were assembled utilizing K metal anode, glass fiber separator, and carbonate solution (0.8 M KPF_6_ in EC/DEC = 1/1 by volume) electrolyte. The cyclic voltammograms (CV) curves of S@P-NCF were recorded at a scan rate of 0.1 mV s^−1^ in the voltage window of 0.5–3.0 V (vs. K^+^/K) (Figure 3a). During the first discharge process, a major peak located at 0.54 V, which corresponds to the reduction from sulfur to KPSs [56,60]. In the following charge process, a broad peak located at 1.71 V (Anodic I) and a weak peak located at 1.97 V (Anodic II), which are related to the depotassiation process and the stepped oxidation reaction of KPSs to sulfur. Specially, the Anodic I peak can be attributed to the oxidation of short-chain KPSs to long-chain KPSs, and the Anodic II peak is ascribed to the final formation of sulfur species [60]. In addition, the CV curve of the initial discharge process is different from the following cycles, which may be attributed to the formation of a cathode electrolyte interphase (CEI) during the initial discharge process [61]. In the subsequent cycles, the cathodic peak located at 0.54 V is replaced by three peaks located at 1.64 (Cathodic I), 1.24 (Cathodic II), and 0.77 V (Cathodic III). This suggests that a multistep conversion of sulfur species occurs during the potassiation process [57]. In addition, there is no obvious difference in the following CV curves, indicating the outstanding reversibility. The counterpart CV curves of S@NCF are shown in Figure S6. Obviously, the voltage hysteresis between the peak Anodic I and the peak Cathodic III of S@NCF (1.05 V) is larger than that of S@P-NCF (0.94 V). Figure 3b displays the first three discharge/charge profiles of S@P-NCF and S@NCF at 0.1 C (1 C = 1675 mAh g^−1^) within the voltage window of 0.5–3.0 V. After the first cycle, two long discharge plateaus at ~1.5 V and ~0.8 V and a long charge plateau at ~1.6 V can be observed, which are consistent with the CV results. Most importantly, S@P-NCF exhibits an ultrahigh initial reversible capacity (1470 mAh g^−1^), much higher than S@NCF (850 mAh g^−1^). The above-mentioned results indicate that richer micropores and larger polar surface area enhance the interaction between sulfur and carbon matrix, improve the utilization and conversion of sulfur, and reduce the polarization of S@P-NCF.

The rate capabilities of sulfur cathodes were measured at current densities from 0.2 to 2 C (Figure 3c). The S@P-NCF delivers reversible capacities of 1430, 1170, 950, 755, and 560 mAh g^−1^ at 0.2, 0.3, 0.5, 1, and 2 C, respectively. On the contrary, S@NCF only delivers capacities of 680 and 400 mAh g^−1^ at current densities of 0.2 and 2 C, respectively, much lower than that of S@P-NCF. However, at small current densities S@P-NCF exhibits a more rapid capacity decay than S@NCF. Figure 3d shows the corresponding discharge/charge profiles of S@P-NCF under various current densities, which reveal much lower polarization potentials, compared to S@NCF (Figure S7). As illustrated in Figure 3e, compared with recently reported KSBs, the rate performance of our S@P-NCF is among the best to date [56,57,62,63,64,65,66,67,68,69,70,71]. Figure 3f shows the cycling performances of S@P-NCF and S@NCF at the current density of 0.2 C. The S@P-NCF exhibits a much higher specific capacity, with an initial reversible capacity of 1470 mAh g^−1^. After 100 cycles, the remaining capacity is 832 mAh g^−1^ and the Coulombic efficiency is ~100%. S@NCF only delivers an initial reversible capacity of 690 mAh g^−1^ and remaining capacity of 556 mAh g^−1^ after 100 cycles. The higher specific capacity of S@P-NCF demonstrates that more abundant micropores and larger polar surface area can enhance the interaction between sulfur and carbon matrix, and thus promote the conversion of sulfur species and realize higher utilization of sulfur. In addition, during the cycling process, the rich mesopores provide ample space for the volume expansion of sulfur species [72]. As shown in Figure 3g, long-term cycling performances of S@P-NCF and S@NCF were performed at a current density of 2 C. After 300 cycles at a current density of 2 C, S@P-NCF delivers a higher capacity retention of 39.0% and a lower capacity loss of 0.13% each cycle. In contrast, S@NCF shows rapid capacity decay during the 50–100 cycles and operates at the low value of <200 mAh g^−1^. The electrochemical performance of S@P-NCF-based K-S batteries under high areal loading conditions has also been attempted to be measured. Figure S8 shows the first five discharge/charge profiles of S@P-NCF with sulfur areal loading of 2.0 mg cm^−2^ at 0.1 C. Of interest is that S@P-NCF delivers a high initial reversible capacity (1100 mAh g^−1^) even at a high sulfur areal loading of 2.0 mg cm^−2^. To further demonstrate the stable long-term cycling performance of the electrodes, the cell impedances of S@P-NCF and S@NCF in the charged states after the 1st and 100th cycles were analyzed by electrochemical impedance spectroscopy (EIS) (Figure 3h and Figure S9). The semicircle at high frequency corresponds to the charge transfer resistance (*R*_ct_) through the electrode/electrolyte interface. The equivalent circuit model and fitted electrode resistance data are shown in Figure 3h and Table S2, respectively. Obviously, S@NCF shows a larger increment of *R*_ct_ from 1411 Ω (1st) to 4901 Ω (100th), compared to S@P-NCF from 383.7 to 1429 Ω. The lower *R*_ct_ of S@P-NCF after the first cycle indicates that porosity engineering and polar nitrogen functionalities provide the fast ion transport and excellent electrical conductivity, which contribute to sulfur utilization and conversion. In addition, the smaller *R*_ct_ increment of S@P-NCF after 100 cycles indicates the superior cycling stability and structural stability of S@P-NCF. The above electrochemical tests show that S@P-NCF has higher specific capacity, sulfur utilization, and better long-term cycling performance at a high current density than S@NCF. Obviously, the ultrahigh initial reversible capacity of S@P-NCF is related to the tight binding between sulfur in the micropores and carbon matrix, which effectively increases the utilization of sulfur. In addition, the larger polar surface area, higher content of pyridinic-/pyrrolic-N, and more abundant mesopores of P-NCF also make an essential contribution to the high capacity and excellent cycling performance.

**Figure 4:**
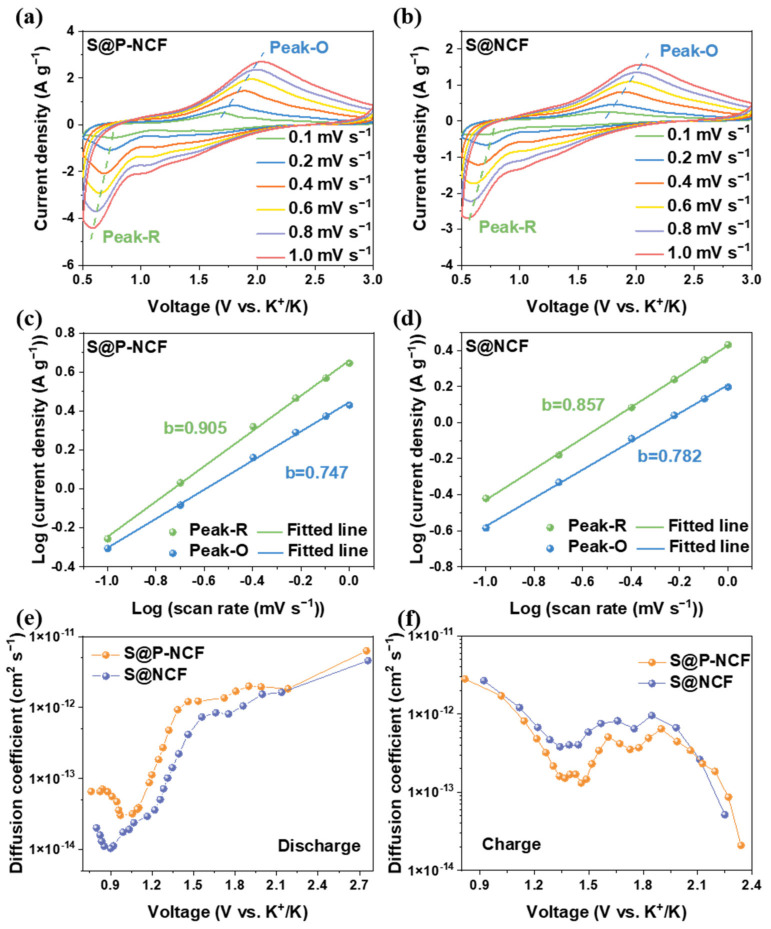
CV curves of (**a**) S@P-NCF and (**b**) S@NCF at different scan rates from 0.1 to 1 mV s^−1^. The linear relation of peak currents and scan rates of (**c**) S@P-NCF and (**d**) S@NCF. The K-ion diffusion coefficient as a function of the states of the (**e**) discharge process and (**f**) charge process.

Multi-scan rates CVs and the galvanostatic intermittent titration technique (GITT) were employed to demonstrate the kinetics of the sulfur cathodes. The CVs at different scan rates were further examined for the in-depth kinetic analyses. Figure 4a,b display the CV curves at the scan rates of 0.1, 0.2, 0.4, 0.6, 0.8, and 1.0 mV s^−1^ of S@P-NCF and S@NCF, respectively. These CV curves exhibit a similar profile, except for a noticeable shift as the scan rate increases. As the scan rate increases, two major peaks (labeled Peak-R and Peak-O, respectively) for both S@P-NCF and S@NCF can be observed. The reaction mechanisms of S@P-NCF and S@NCF can be evaluated based on an established formula of log *i* = *b* log *v* + log *a*, where *i* is the peak current, *v* is the scan rate of the CV and *b* is a constant ranging from 0.5 to 1. When the *b* value is approaching 0.5 or 1, the system is under the diffusion dominated or surface capacitive-controlled process, respectively [73]. Figure 4c shows the *b* values of S@P-NCF for Peak-R and Peak-O, which are fitted to be 0.905 and 0.747, respectively. This indicates that the reduction conversion reaction of S@P-NCF is a rapid dynamic pseudocapacitance behavior. The *b* values of S@NCF for Peak-R and Peak-O are 0.857 and 0.782, respectively (Figure 4d). Interestingly, for S@P-NCF, the *b* value of Peak-R is higher than that of S@NCF, while the *b* value of Peak-O is lower than that of S@NCF. This indicates that S@P-NCF exhibits more facile discharge kinetics, while for the charge process, S@NCF shows better reaction kinetics. The GITT was measured to further reveal the K-ion diffusion kinetics. Figure S10 displays the discharge/charge profiles of GITT. Figure 4e,f show the K-ion diffusion coefficients under different voltages calculated based on Figure S11 during discharge and charge process, respectively. As shown in Figure 4e, in the discharge process, S@P-NCF delivers higher K-ion diffusion coefficients than those of S@NCF. Nonetheless, in the charge process, S@NCF has higher K-ion diffusion coefficients (Figure 4f). This indicates that the reaction kinetics of S@P-NCF is better in the discharge process and inferior during the charge process than that of S@NCF, which is consistent with the results in Figure 4c,d. It is reasonable to believe that more microporous structures and nitrogen heteroatoms in P-NCF can enhance the binding between sulfur and carbon matrix, improve the reaction kinetics during the discharge process, and accelerate the conversion of sulfur species to K_2_S, thus, achieving an ultrahigh specific capacity and large utilization of sulfur. Nevertheless, during the charge process, the depotassiation of low-order KPSs is more sluggish for S@P-NCF, which makes insoluble and insulating low-order KPSs easy to accumulate to form “dead polysulfide”, and thus lose electroactivity, leading to capacity decay and pessimistic capacity retention [28].

**Figure 5:**
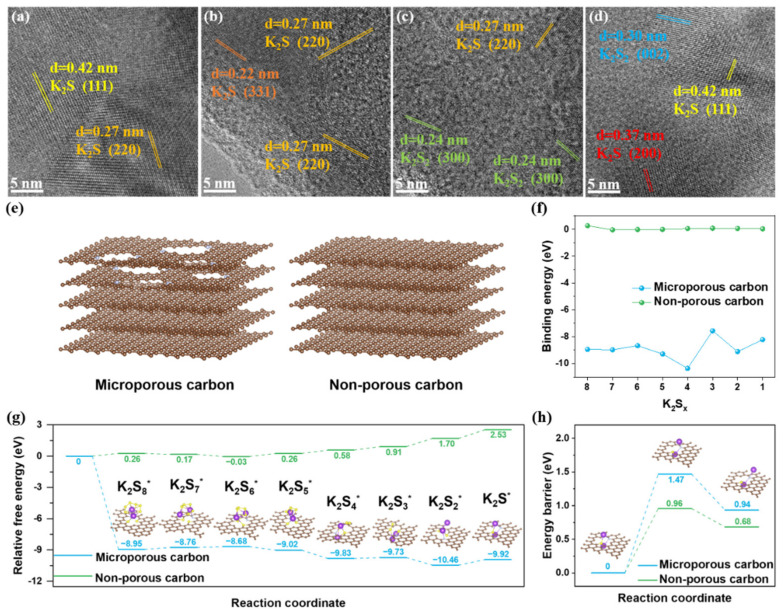
HRTEM images of (**a**,**b**) S@P-NCF and (**c**,**d**) S@NCF discharged to 0.5 V versus K^+^/K. (**e**) Schematic illustration of microporous carbon model and non-porous carbon models. (**f**) Comparison of the binding energies of various K_2_S_x_ molecules bound to microporous and non-porous carbon models, respectively. (**g**) Energy profiles for the reduction in KPSs on microporous and non-porous carbon models; the insets are the corresponding adsorption configurations of KPSs on microporous carbon model. (**h**) The decomposition energy profiles of K_2_S on microporous and non-porous carbon models; the insets are decomposition path of K_2_S on microporous carbon model.

HRTEM was performed to investigate the origin of the ultrahigh capacity for S@P-NCF. From the state of potassiation to 0.5 V for S@P-NCF, the HRTEM images clearly show that some nanosized crystals embed in the carbon matrix (Figure 5a,b), which are ascribed to the K_2_S phase. For S@NCF, a lot of K_2_S_2_ nanocrystals can be observed in the HRTEM images (Figure 5c,d). The SAED images (Figure S12) also demonstrate the difference. As shown in Figure S12a, the diffraction rings are ascribed to the (220), (222), (400) planes of K_2_S phase for S@PNCF. For S@NCF there are clear diffraction spots attributed to K_2_S_2_ in the SAED image (Figure S12b). Different discharge products determine the number of electrons involved in the reaction process. S@P-NCF reaching K_2_S in the discharge product displays much higher capacity. In contrast, for S@NCF, kinetics being more sluggish during the discharge process leads to an earlier end of the discharge process with a large amount of K_2_S_2_ in the discharge product. Therefore, porosity engineering can enhance the kinetics during the discharge process and accomplish a deeper discharge, thereby enabling higher capacity for the cathode.

In order to gain atomistic insight into the mechanism of the interaction between KPSs and micropores, DFT simulations were performed to determine the role of micropores in the adsorption and conversion of KPSs. As shown in Figure 5e, carbon defects on the nitrogen-doped graphene layer are used to simulate the micropores, and the pore-free graphene model is used as a comparison. The optimized configurations of various KPSs adsorbed on microporous carbon and graphene are displayed in Figure S13. As shown in Figure 5f, the binding energy of KPSs on microporous carbon is significantly more negative compared to the non-porous model. In general, the strength of adsorption increases as the binding energy becomes more negative. This indicates that the microporous carbon is more effective in adsorbing KPSs. As shown in Figure 5g, the Gibbs free energies for all reaction steps from sulfur to K_2_S during the discharge process were calculated. Overall, the reaction from sulfur to K_2_S is spontaneously exothermic on microporous carbon but endothermic on non-porous model. This suggests that the discharge process is more thermodynamically favorable with the influence of the micropores. On the contrary, because the discharge process on graphene is endothermic, this means that the reaction from K_2_S to sulfur is spontaneously exothermic on the non-porous model. This suggests that the charge process on graphene is thermodynamically favorable without the influence of micropores. Moreover, the K_2_S decomposition barriers on microporous and non-porous carbon models were investigated (Figure 5h). Obviously, both the dissociation energy and the decomposition barrier of K_2_S are higher on microporous carbon. This indicates that the micropore plays an inhibitory role in the decomposition of K_2_S and is unfavorable to the oxidation of K_2_S during the charge process. DFT simulation results show that micropores have a strong adsorption to KPSs, which is favorable to the discharge process. Nonetheless, on the other side, the decomposition of K_2_S becomes difficult, which is detrimental to the charge kinetics.

## 3. Conclusions

In this report, we employ a porosity engineering strategy to a nitrogen-rich carbon foam to construct a sulfur host for high-performance room temperature K-S batteries. The abundant microporosity greatly enlarges the polar surface area of the carbon host, thereby reinforcing the affinity of sulfur species on the electrode, both at the pristine state and during electrochemical cycling. Meanwhile, the mesoporosity create ample room to buffer the large volume change of sulfur upon repeated potassiation. Ultra-high capacity of 1470 mAh g^−1^ at 0.1 C was obtained with 560 mAh g^−1^ remaining at high rate of 2 C, which is superior to most state-of-the-art KSB performances to date. Both electrochemical analysis (GITT and multi-rate CVs) and DFT calculation demonstrate that the microporosity with nitrogen functionalities contribute to the sulfur reduction for the end member of K_2_S, which nonetheless deteriorates the oxidation (charge) kinetics by increasing the energy barrier of K_2_S decomposition.

## Data Availability

All relevant data are included within the article and its Supporting Information files.

## Supplementary Materials

The following supporting information can be downloaded at: https://www.mdpi.com/article/10.3390/nano12223968/s1, Figure S1: (a) SEM and (b,c) TEM images of the NCF (inset: the corresponding SAED image); Figure S2: (a) SEM and (b,c) TEM images of the S@NCF (inset: the corresponding SAED image); Figure S3: I_D_/I_G_ values of P-NCF, S@P-NCF, NCF, and S@NCF calculated from the Raman spectra; Figure S4: (a) XPS survey spectra of S@P-NCF and S@NCF. High-resolution XPS spectra of (b) S 2p and (c) C 1s for S@NCF, and (d) N 1s for NCF; Figure S5: Distribution of three nitrogen species (pyridinic-N, pyrrolic-N, and graphitic-N) in the P-NCF and NCF; Figure S6: CV of the S@NCF at a scan rate of 0.1 mV s^−1^; Figure S7: Galvanostatic discharge/charge profiles of S@NCF at various current densities; Figure S8: Galvanostatic discharge/charge profiles of S@P-NCF with sulfur areal loading of 2.0 mg cm^−2^; Figure S9: EIS Nyquist plots and corresponding fitted lines of the S@P-NCF and S@NCF at different cycles; Figure S10: GITT profiles of the (a) discharge and (b) charge processes measured at the current density of 0.03 C; Figure S11: Schematic diagram of the parameters in the GITT curve used to calculate the diffusion coefficient: (a) discharge process and (b) charge process. (IR drop is the voltage change that occurs when the charge/discharge process and the relaxation switch with each other); Figure S12: SAED images of (a) S@P-NCF and (b) S@NCF discharged to 0.5 V versus K^+^/K; Figure S13: Detailed adsorption atomic configuration for KPSs on microporous carbon and non-porous carbon; Table S1: The surface area and pore volume of the as-prepared samples; Table S2: Electrode resistance obtained from the equivalent circuit. References [74,75,76,77,78,79,80,81] were cited in the Supplementary Materials.
