# Supplementary material for: Porosity Engineering towards Nitrogen-Rich Carbon Host Enables Ultrahigh Capacity Sulfur Cathode for Room Temperature Potassium–Sulfur Batteries
Source: Nanomaterials (Basel). 2022 Nov 10;12(22):3968. doi: 10.3390/nano12223968 (PMC9696911; doi:10.3390/nano12223968)
Supplement: Supplementary file 1 [file nanomaterials-12-03968-s001.zip › nanomaterials-1950304-supplementary.pdf]

Supplementary Materials

Experimental Section

Materials synthesis

Synthetic procedures for graphitic carbon nitride (g-C_3N_4). Typically, 20 g of urea was put in a ceramic container with a cover and heated under static air at 550 ℃ for 2 h with a ramping rate of 5 ℃ min^{-1}. The resultant yellow powder was collected without further treatment and denoted as g-C_3N_4.

Synthetic procedures for starch xerogel. Typically, 0.5 g of g-C_3N_4 was dispersed uniformly in 40 mL of deionized H_2O, which was denoted as solution A. The solution A was heated to 100 ℃ and stirred at ~500 rpm. 2 g starch was dispersed in 10 mL of deionized H_2O, forming a turbid solution, which was denoted as solution B. The solution B was added to the solution A at 100 ℃ with stirring. The mixed solution gradually thickened. After cooling to room temperature, the hydrogel was obtained. Then the starch xerogel was obtained after freeze drying.

Porosity engineering strategy towards nitrogen-rich carbon foam (P-NCF). After being annealed at 900 ℃ for 2h in an argon atmosphere at a ramping rate of 5 ℃ min^{-1}, the nitrogen-rich carbon foam (NCF) was obtained. Then the as-synthesized NCF was further treated at 800 ℃ for 1h in a carbon dioxide atmosphere at a ramping rate of 5 ℃ min^{-1}, the P-NCF was obtained.

Synthetic procedures for S@P-NCF and S@NCF. A mixture of P-NCF and sulfur powder with a mass ratio of 1:2 was ground by mortar. Then the mixture

was vacuum-sealed in a glass tube and heated at 500 ℃ for 6 h at a ramping rate of 5 ℃ min^{-1}. Afterwards, the resultant mixture was further annealed at 200 ℃ for 2 h in the flowing argon to remove the residual sulfur on the surface of P-NCF, and S@P-NCF was obtained. S@NCF was synthesized via the same procedure by utilizing NCF instead of P-NCF.

Material Characterizations

The microstructure and surface morphology were observed using a thermal field emission scanning electron microscope (SEM, JSM-7800F, JEOL) and a transmission electron microscope (TEM, JEM-2100F, JEOL). X-ray diffraction (XRD) patterns were collected with Cu Kα radiation (λ = 1.54056 Å) at 40 kV and 40 mA on a D8 Advance, Bruker diffractometer. The chemical and electronic states of the surface were analyzed by X-ray photoelectron spectroscopy (XPS, Ultra^{DLD}, KRATOS). Raman spectra were recorded with a 532 nm excitation source on an inVia reflex, RENISHAW. The N_2 adsorption−desorption isotherm measurements (APSP 2460, Micromeritics) were performed to analyze the specific surface area and the pore size distribution feature based on multipoint Brunauer−Emmett−Teller (BET) theory. Before N_2 adsorption−desorption isotherm measurements, P-NCF and NCF were degassed under vacuum at 200 ℃ for 8 h and S@P-NCF and S@NCF were degassed under vacuum at room temperature for 12 h. For all materials, the analysis temperature for the N_2 adsorption-desorption isotherm measurements was -196 ℃. The sulfur contents of S@C composites were calculated according

to thermogravimetric analysis (TGA, SDT650, TA) carried out under flowing N_2 with a heating rate of 10 ℃ min^{-1}.

Electrochemical Measurements

To fabricate working electrodes, active materials (S@P-NCF/S@NCF, 70 wt %), were mixed with multiwalled carbon nanotubes (MWCNT, 20 wt %), and poly(vinylidene difluoride) (PVDF, 10 wt %) in *N*-methyl-2-pyrrolidinone (NMP) to form a slurry. The slurry was casted onto the aluminum foil and dried at 60 ℃ overnight in a vacuum oven. The sulfur areal loading is ~0.8 mg cm^{-2} for both S@P-NCF cathode and S@NCF cathode. Commercial potassium metal was used as counter electrodes. Glass fiber/C (GF/C, Whatman) membrane was used as separator. A solution of 0.8 M potassium hexafluorophosphate (KPF_6) in ethylene carbonate/diethyl carbonate (EC/DEC = 1/1 by volume, 100 μL) was used as the electrolyte. The CR2032 coin cells were assembled in a glovebox (argon atmosphere, O_2 and H_2O levels < 0.1 ppm). The charge/discharge measurements were performed in the voltage window from 0.5 to 3.0 V (vs K^+/K) on LAND and NEWARE battery test systems. The cyclic voltammetry (CV) and electrochemical impedance spectroscopy (EIS) measurements (100 k–0.01 Hz) were conducted in an electrochemical workstation (CHI 660E). Capacity calculation was based on the mass of sulfur.

Theoretical calculations

All the calculations were performed within the framework of the density functional theory (DFT) as implemented in the Vienna Ab initio Software

Package (VASP 5.4.4) code within the Perdew-Burke-Ernzerhof (PBE) generalized gradient approximation and the projected augmented wave (PAW) method [1-4]. The cutoff energy for the plane-wave basis set was set to 450 eV. The Brillouin zone of the surface unit cell was sampled by Monkhorst-Pack (MP) grids for catalyst structure optimizations [5]. A 5 × 3 supercell of the graphene surface was constructed to model the carbon host in this work. The microporous and non-porous carbon models were determined by 3 × 3 × 1 Monkhorst-Pack grid. The convergence criterion for the electronic self-consistent iteration and force was set to 10^{-5} eV and 0.01 eV/Å, respectively. A vacuum layer of 15 Å was introduced to avoid interactions between periodic images.

The free energies of adsorbates and transition states at temperature T were estimated according to the harmonic approximation, and the entropy is evaluated using the following equation:

$$S(T) = k_B \sum_{i}^{harm\,DOF} \left[\frac{\varepsilon_i}{k_B T(e^{\frac{\varepsilon_i}{k_B T}} - 1)} - \ln(1 - e^{-\frac{\varepsilon_i}{k_B T}}) \right]$$

where k_B is Boltzmann's constant and DOF is the number of harmonic energies (ε_i) used in the summation denoted as the degree of freedom, which is generally 3N, where N is the number of atoms in the adsorbates or transition states. Meanwhile, the free energies of gas phase species are corrected as:

$$G_g(T) = E_{elec} + E_{ZPE} + \int C_p dT - TS(T)$$

where C_p is the gas phase heat capacity as a function of temperature derived from Shomate equations and the corresponding parameters in the equations

were obtained from NIST.

Transition states were located using the climbing image nudged elastic band (CI-NEB) method and each transition state was confirmed to have a single imaginary vibrational frequency along the reaction coordination [6].

Supplementary Figures and Tables

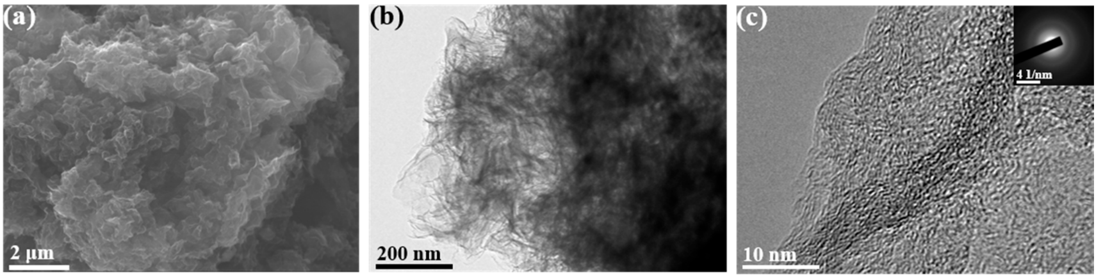

Figure S1. (a) SEM and (b, c) TEM images of the NCF (inset: the corresponding SAED image).

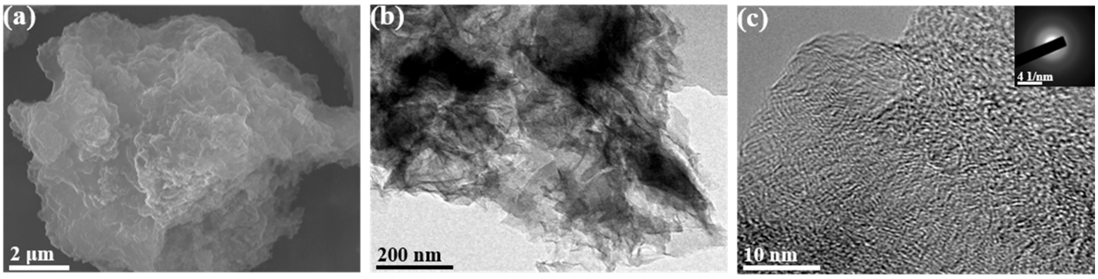

Figure S2. (a) SEM and (b, c) TEM images of the S@NCF (inset: the corresponding SAED image).

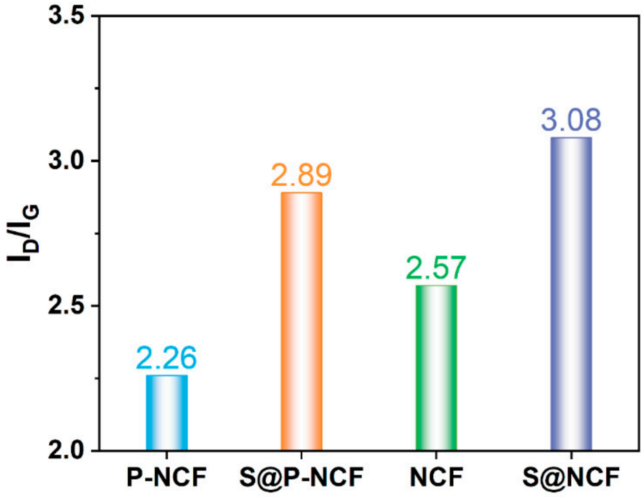

Figure S3. I_D/I_G values of P-NCF, S@P-NCF, NCF, and S@NCF calculated from the Raman spectra.

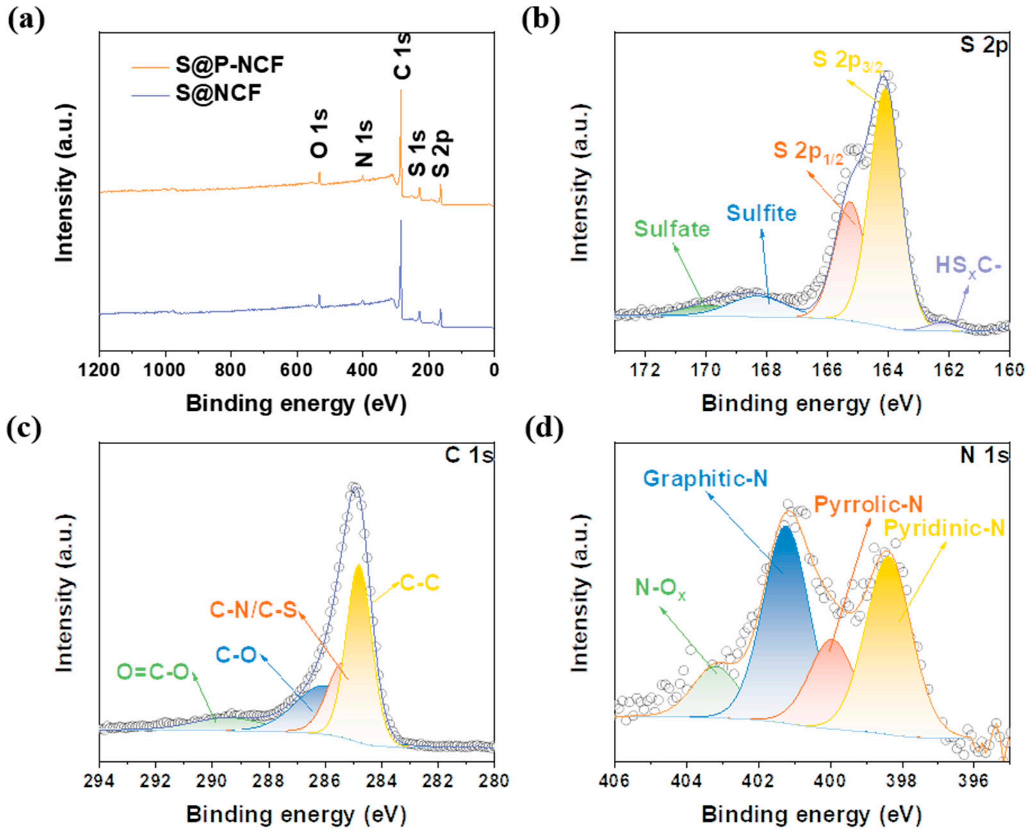

Figure S4. (a) XPS survey spectra of S@P-NCF and S@NCF. High-resolution XPS spectra of (b) S 2p and (c) C 1s for S@NCF, and (d) N 1s for NCF.

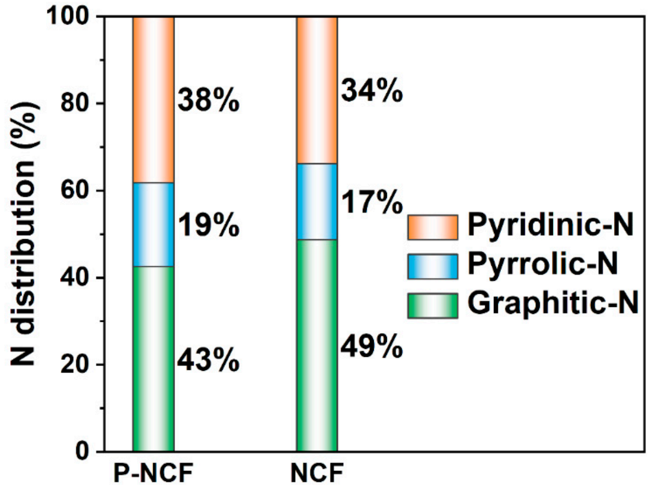

Figure S5. Distribution of three nitrogen species (pyridinic-N, pyrrolic-N, and graphitic-N) in the P-NCF and NCF.

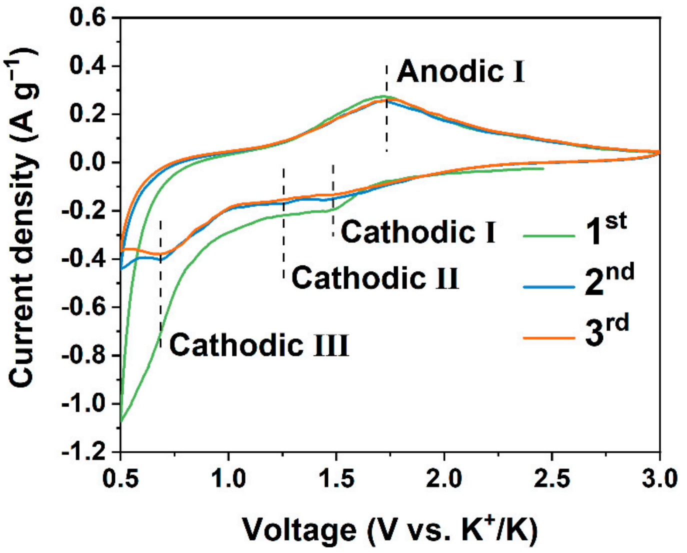

Figure S6. CV of the S@NCF at a scan rate of 0.1 mV s^{-1}.

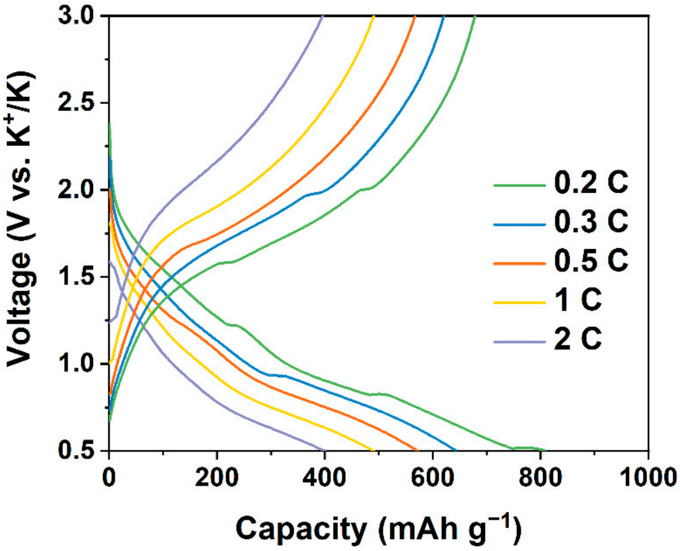

Figure S7. Galvanostatic discharge/charge profiles of S@NCF at various current densities.

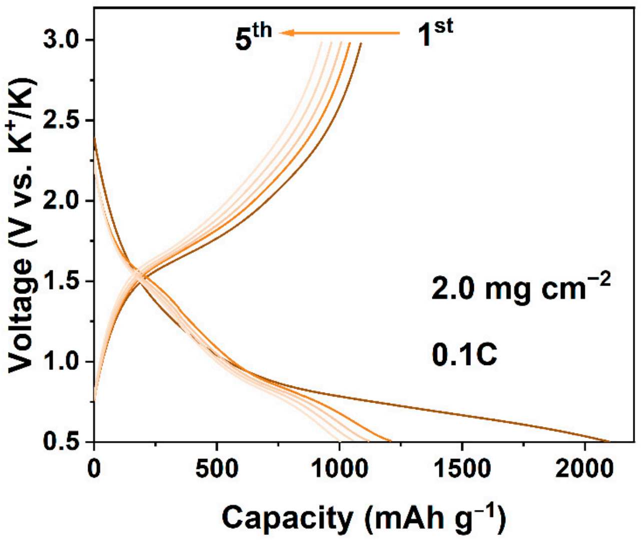

Figure S8. Galvanostatic discharge/charge profiles of S@P-NCF with sulfur areal loading of 2.0 mg cm^{-2}.

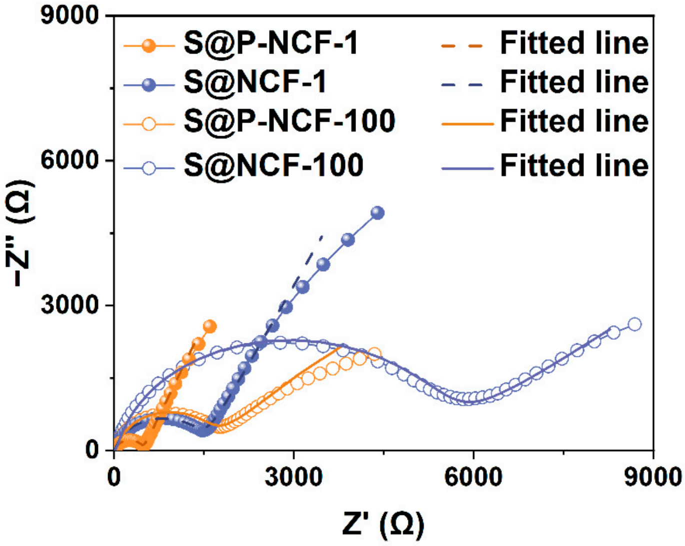

Figure S9. EIS Nyquist plots and corresponding fitted lines of the S@P-NCF and S@NCF at different cycles.

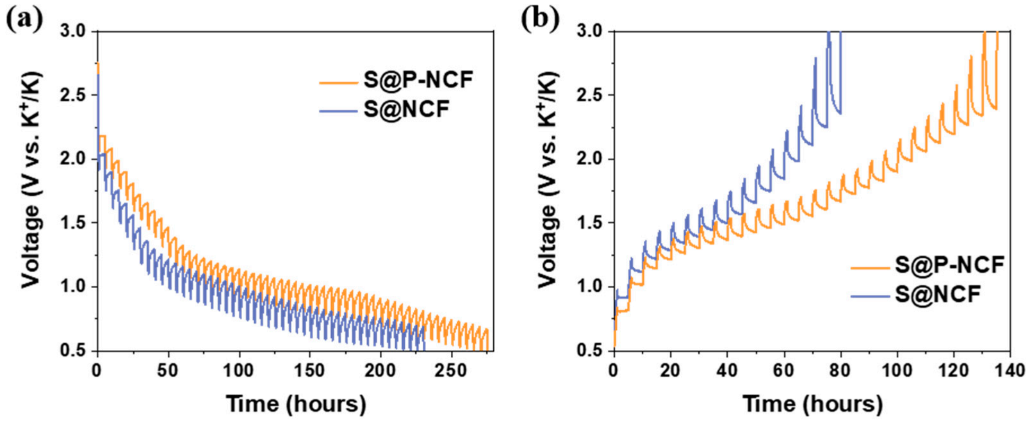

Figure S10. GITT profiles of the (a) discharge and (b) charge processes measured at the current density of 0.03 C.

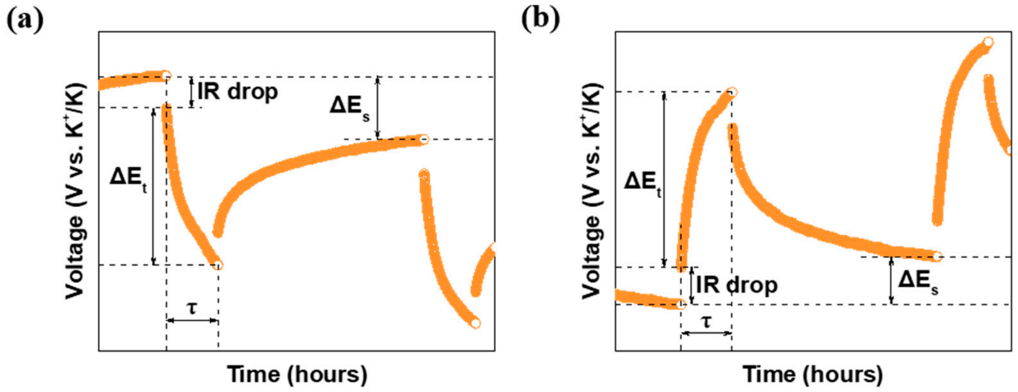

Figure S11. Schematic diagram of the parameters in the GITT curve used to calculate the diffusion coefficient: (a) discharge process and (b) charge process. (IR drop is the voltage change that occurs when the charge/discharge process and the relaxation switch with each other).

The current pulse with the discharge/charge process for 1 h at 0.03 C and subsequent relaxation for 4 h. The K-ion diffusion coefficients under different voltages can be calculated via the following equation: $D = \frac{4}{\pi\tau}\left(\frac{m_B V_M}{M_B S}\right)^2\left(\frac{\Delta E_s}{\Delta E_t}\right)^2$, where D is the K^+ diffusion coefficient, τ is the constant current pulse time, m_B is the mass of the active material, V_M is the molar volume of the active material, M_B is the molar mass of the active material, M_B/V_M is approximated as the density of the active material, S is the area of the electrode, ΔE_s is the voltage charge of the steady state voltage, ΔE_t is the voltage charge during a constant pulse (ΔE_s and ΔE_t are defined as shown in Figure S9) [7,8].

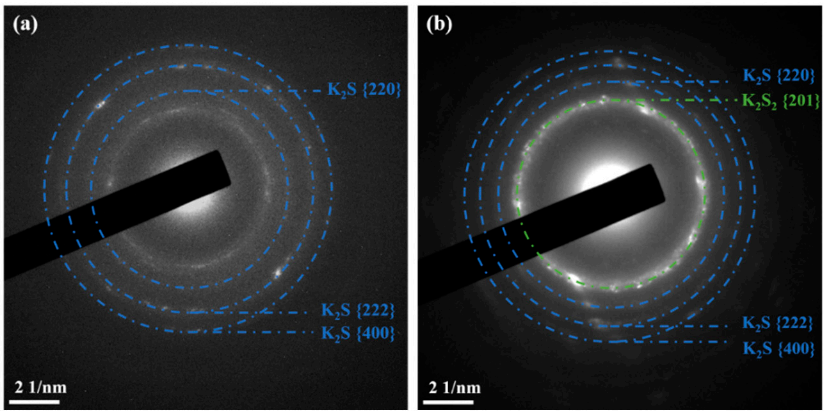

Figure S12. SAED images of (a) S@P-NCF and (b) S@NCF discharged to 0.5 V versus K^+/K.

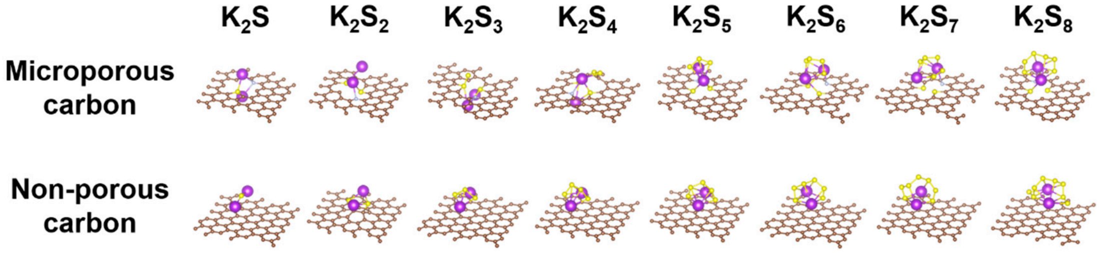

Figure S13. Detailed adsorption atomic configuration for KPSs on microporous carbon and non-porous carbon.

Table S1. The surface area and pore volume of the as-prepared samples.

	Micropore area (m^2 g^{-1})	BET surface area (m^2 g^{-1})	Total pore volume (cm^3 g^{-1})	Micropore volume (cm^3 g^{-1})
P-NCF	754.4427	1167.4092	1.0028	0.3388
NCF	516.1193	619.9528	0.4506	0.1363
S@P-NCF	3.4429	44.9297	0.1707	0.0016
S@NCF	1.5503	33.2649	0.1371	0.0005

Table S2. Electrode resistance obtained from the equivalent circuit.

	S@P-NCF-1	S@NCF-1	S@P-NCF-100	S@NCF-100
R_0(Ω)	4.25	3.42	13.14	8.66
R_{ct} (Ω)	383.7	1411	1429	4901

References

1. Perdew, J.P.; Burke, K.; Ernzerhof, M. Generalized Gradient Approximation Made Simple. *Phys. Rev. Lett.* **1996**, *77*, 3865-3868, doi:10.1103/PhysRevLett.77.3865.

2. Hammer, B. Improved adsorption energetics within density-functional theory using revised Perdew-Burke-Ernzerhof functionals. *Phys. Rev. B* **1999**, *59*, 7413-7421, doi:10.1103/PhysRevB.59.7413.

3. Blochl, P.E. Projector augmented-wave method. *Phys. Rev. B* **1994**, *50*, 17953-17979, doi:10.1103/physrevb.50.17953.

4. Kresse, G.; Joubert, D. From ultrasoft pseudopotentials to the projector augmented-wave method. *Phys. Rev. B* **1999**, *59*, 1758-1775,

doi:10.1103/PhysRevB.59.1758.

5. Monkhorst, H.J.; Pack, J.D. Special points for Brillouin-zone integrations. *Phys. Rev. B* **1976**, *13*, 5188-5192, doi:10.1103/PhysRevB.13.5188.

6. Behler, J. A climbing image nudged elastic band method for finding saddle points and minimum energy paths. *J. Chem. Phys.* **2000**, *113*, 9901-9904, doi:10.1063/1.3553717.

7. Dees, D.W.; Kawauchi, S.; Abraham, D.P.; Prakash, J. Analysis of the Galvanostatic Intermittent Titration Technique (GITT) as applied to a lithium-ion porous electrode. *Journal of Power Sources* **2009**, *189*, 263-268, doi:10.1016/j.jpowsour.2008.09.045.

8. Ma, Q.; Zhong, W.; Du, G.; Qi, Y.; Bao, S.J.; Xu, M.; Li, C. Multi-step Controllable Catalysis Method for the Defense of Sodium Polysulfide Dissolution in Room-Temperature Na-S Batteries. *ACS Appl. Mater. Interfaces* **2021**, *13*, 11852-11860, doi:10.1021/acsami.0c21267.
